# In Silico Exploration of Microtubule Agent Griseofulvin and Its Derivatives Interactions with Different Human β-Tubulin Isotypes

**DOI:** 10.3390/molecules28052384

**Published:** 2023-03-05

**Authors:** Parisa Aris, Masoud Mohamadzadeh, Alibek Kruglikov, Mahbubeh Askari Rad, Xuhua Xia

**Affiliations:** 1 Department of Biology, University of Ottawa, 30 Marie Curie, Station A, P.O. Box 450, Ottawa, ON K1N 6N5, Canada; 2 Department of Chemistry, Faculty of Sciences, University of Hormozgan, Bandar Abbas 71961, Iran; 3 Ottawa Institute of Systems Biology, Ottawa, ON K1H 8M5, Canada

**Keywords:** β-tubulin isotypes, cancer, docking, griseofulvin, griseofulvin derivatives, molecular dynamics

## Abstract

Tubulin isotypes are known to regulate microtubule stability and dynamics, as well as to play a role in the development of resistance to microtubule-targeted cancer drugs. Griseofulvin is known to disrupt cell microtubule dynamics and cause cell death in cancer cells through binding to tubulin protein at the taxol site. However, the detailed binding mode involved molecular interactions, and binding affinities with different human β-tubulin isotypes are not well understood. Here, the binding affinities of human β-tubulin isotypes with griseofulvin and its derivatives were investigated using molecular docking, molecular dynamics simulation, and binding energy calculations. Multiple sequence analysis shows that the amino acid sequences are different in the griseofulvin binding pocket of βI isotypes. However, no differences were observed at the griseofulvin binding pocket of other β-tubulin isotypes. Our molecular docking results show the favorable interaction and significant affinity of griseofulvin and its derivatives toward human β-tubulin isotypes. Further, molecular dynamics simulation results show the structural stability of most β-tubulin isotypes upon binding to the G1 derivative. Taxol is an effective drug in breast cancer, but resistance to it is known. Modern anticancer treatments use a combination of multiple drugs to alleviate the problem of cancer cells resistance to chemotherapy. Our study provides a significant understanding of the involved molecular interactions of griseofulvin and its derivatives with β-tubulin isotypes, which may help to design potent griseofulvin analogues for specific tubulin isotypes in multidrug-resistance cancer cells in future.

## 1. Introduction

Griseofulvin is the secondary metabolite of several fungal species. Since its first isolation from *Penicillium griseofulvum* in 1939 [1], it has been used to treat dermatophyte infections [2]. The biosynthetic griseofulvin gene cluster including 13 *gsf* genes responsible for producing griseofulvin was first identified in *Penicillium aetiopicum* through shotgun sequencing and genome mining over a decade ago [3]. In addition to *Penicillium*, other genera of ascomycetes, including *Aspergillus alliaceus*, *Xylaria flabelliformis*, and *Memoniella echinata*, have shown the ability to produce griseofulvin as they harbor the *gsf* gene cluster in their genomes [4,5,6]. However, our recent study showed that only 7 out of 13 *gsf* genes might be essential for griseofulvin biosynthesis [7].

Mechanistically, griseofulvin stabilizes microtubule dynamics and mediates microtubule abnormalities such as misaligned chromosomes and multipolar spindles. These abnormalities suppress cell division dramatically and lead to a cell-death response [8]. Additionally, increased p53 accumulation in the nucleus of these cells revealed that they were undergoing apoptosis [9]. A recent research reported the binding potential of griseofulvin with keratin cytoskeletons intermediate filaments proteins K8 and K18, which results in changing the keratin solubility and formation of Mallory bodies (MBs) in hepatocytes [10]. Occasional hepatitis and Mallory bodies formation in hepatocytes has been reported in humans and rodents after griseofulvin treatment [11,12,13], although severe hepatitis is generally observed in rodents, which can be explained by the stronger griseofulvin binding affinity to K18 in rodents than in humans [10]. 

Griseofulvin has drawn increasing attention in recent years because of its potential to suppress the replication of the hepatitis C virus (HCV) [14], and most recently for its repurposing potential against SARS-CoV-2 [15]. Interestingly, griseofulvin is currently being employed as a therapeutic agent in cancer treatment studies including breast and colon cancer [8,9]. The major category of anticancer drugs are tubulin binding agents (TBAs) that directly target β-tubulins to suppress spindle microtubule dynamics, thus disrupting cell division and inducing cell death [16,17]. There are distinct mammalian β-tubulin isotypes produced by a multigene family which encodes different proteins with diverse tissue expressions and distributions [18]. The amino-terminal (N-terminal) and intermediate domains of tubulin isotypes are highly conserved among different species, but they can be distinguished at their carboxy-terminal (C-terminal) tails, which facilitate interactions with proteins and serve as sites for post-translational modifications [19,20]. The expression of tubulin isotypes is disturbed in cancer cells compared to the healthy tissue. Notably, anomalous tubulin isotype expression corresponds with patient outcome and therapy response [21]. Clinical specimens analysis revealed the elevated expression of multiple tubulin isotypes, including βI, βII, βIII, βIVa, and βV, are significantly associated with aggressive clinical behavior, multidrug-resistant cancer, and unfavorable patient outcomes [21,22,23]. Additionally, it has been found that βII, βIII, and βIV tubulin isotypes show different binding affinities for anticancer drugs, such as taxol, colchicine, and nocodazole [24,25,26]. Therefore, these drug-resistant tubulin isotypes have been identified as promising targets for developing novel anticancer drugs.

Griseofulvin is a potent microtubule agent whose binding site overlaps with taxol, a cancer chemotherapy drug, at the β-tubulin H6–H7 loop [27]. However, the molecular mechanism of interactions and the binding affinities with different β-tubulin isotypes present in cancer cells are not well understood. Furthermore, the synthesis of novel griseofulvin derivatives has received increasing attention in recent years. An experimental study reported that griseofulvin analogues with a sulfonyl group in 2′ or 3′-position exhibited significant activities against oral squamous carcinoma and a triple-negative breast cancer cell line resistant to microtubule inhibitors [28]. It is also reported that the modification of the 2′-benzyloxy and 2′-(4-methylbenzyloxy) analogues remarkably enhanced the anticancer activity more than 25-fold compared to griseofulvin [29]. 

In this study, we investigated the binding mode, involved molecular interactions, and binding affinities of griseofulvin and its derivatives toward human β-tubulin isotypes by employing molecular approaches; i.e., docking, molecular dynamics simulation, and binding energy calculations. Molecular dynamics simulations were also performed into the best griseofulvin-derivative binding site of β-tubulin isotypes to validate the docking results. Our molecular docking analysis confirms the binding potential of griseofulvin to the β-tubulin isotypes at the taxol site. Furthermore, our virtual screenings and molecular docking results suggest five griseofulvin derivatives with more negative binding energies have favorable binding potential and interactions with β-tubulin isotypes. These findings improved our knowledge of griseofulvin molecular interactions with β-tubulin isotypes and it may suggest some griseofulvin derivatives could be promising in designing future therapeutic options for specific β-tubulin isotypes of tumor cells in patients.

## 2. Results and Discussion

### 2.1. Virtual Screening and Molecular Docking Studies

**Figure 1:**
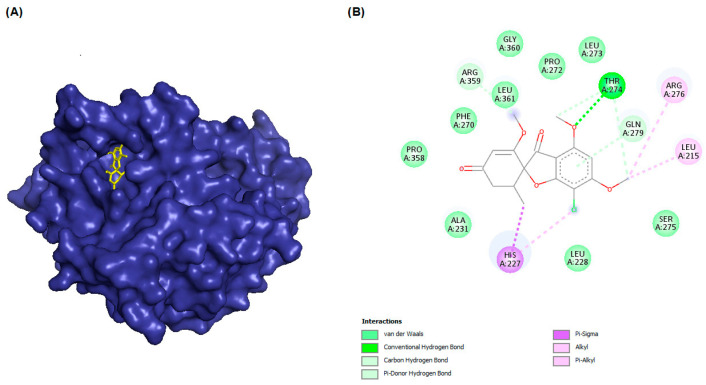
(**A**) The 3D griseofulvin binding pose (yellow), and (**B**) griseofulvin interactions at the active site of TUBB5 (blue). Images were captured by PyMol and Discovery Studio, respectively.

In this study, PyRx was used for virtual screening of ligand candidates to determine the binding modes and affinities toward TUBB5. The PyRx results contain binding energies and RMSDs values for each binding mode of the 464 analogues are shown in Supplementary File S4. To further investigate the ability of binding griseofulvin and its derivatives to tubulin, the binding mode and interactions of griseofulvin and the most active derivatives with the human β-tubulin were examined by molecular docking studies using AutoDock. The 3D structure and docking mode of griseofulvin at the human β-tubulin active site are shown in Figure 1. 

The five derivatives with the lowest binding energy and the highest number of interactions in terms of hydrogen bonds and hydrophobic interactions were selected for further analysis. Table 1 shows two-dimensional (2D) structures of the best-docked ligand molecules, and computed binding energies towards the tubulin protein. The number of rotatable bonds for each molecule was defined by AutoDock. It is expected that molecules with a greater number of rotatable bonds will require a greater number of energy evaluations to converge to an energy minimum, due to a greater number of degrees of freedom and conformational states [30]. Moreover, it is known that increasing the number of rotatable bonds makes it more difficult for Autodock to find the correct docking conformations [31]. Based on our computational predictions, griseofulvin derivatives G1-G5, with the number of rotatable bonds ranging from 3 to 11, show lower binding energies ranging from −8.8 to −9.1 kcal mol^−1^ compared to griseofulvin with a binding energy of −6.6 kcal mol^−1^ with only 3 rotatable bonds. The structure analysis of selected derivatives suggests that functional groups containing oxygen, such as methoxy in G1 and aromatic ether group in derivative G2, provide a better interaction and higher affinity toward receptors. Moreover, structures containing benzene, such as dichlorobenzene in G3, fluoromethyl benzene in G4, and acetate ester group in derivative G5, could enhance the interaction between ligands and proteins. 

Even though binding energy indicates how strong the interaction between ligand and β-tubulin protein could be, Moriguchi octanol-water partition coefficient (MlogP) values were calculated and considered for the drug-diffusion ability into the cells (Table 1). MlogP is a useful structure-based factor developed by Moriguchi et al. [32] to estimate and compare the distribution of drugs within the cells, organs, and the body [33]. As shown in Table 1, griseofulvin shows a lower partition coefficient (MlogP = 0.71) than other compounds, indicating a stronger ability of chemical to distribute through the body and into the cells. However, the MlogP values of griseofulvin derivatives G1-G5 are in good agreement, ranging from 1.96 to 2.98 (Table 1).

As a result, the five derivatives described above, as well as griseofulvin and taxol were then docked into the griseofulvin binding site of nine distinct β-tubulin isotypes. Taxol, an antimicrotubule agent with remarkable antimitotic activity, was used as the control for comparing the binding energies and interactions with tubulin isotypes in this study. Interestingly, griseofulvin-binding sites overlap with the taxol binding site at the beta-tubulin H6–H7 loop [27]. Docking studies revealed that griseofulvin and its derivatives have different binding conformations and energies, depending on the residue composition variations in and around the binding pocket of different β-tubulin isotypes (Table 2).

As shown in the heat map above, the binding energies of five griseofulvin derivatives with binding energies −7.29 to −10.3 kcal mol^−1^ are more negative than taxol and griseofulvin, with binding energies ranging from −6.29 to −8.11 and −6.8 to −7.38 kcal mol^−1^, respectively. The studies exhibited that compounds G1 and G3 are good examples of lower binding energies, greater than −8.09 kcal mol^−1^ towards all β-tubulin isotypes, while for compound G2, interaction with βIIb, βIVa, βIVb, and βV are the strongest binding tubulin isotypes with binding energies −10.3, −9.88, −9.68, and −9.63 kcal mol^−1^, respectively. Compound G5 had the best binding energies when docked to isoforms βV, βIVa, and βIII dominates. However, other derivatives docked well in the griseofulvin-binding site with a binding free energy of at least −7.36 kcal mol^−1^.

The analysis of hydrogen bonds and hydrophobic interactions of griseofulvin derivatives docked with β-tubulin isotypes shows differences in the binding pocket of different β-tubulin, which are listed in Table 3. Worthy of note are griseofulvin and the hydrogen bonds formation of its derivatives with the residues Thr274; and Gln279 of β-tubulin isotypes IIa, IIb, III, IVa, IVb, and VIII. More hydrogen-bond interaction can be observed between griseofulvin and derivatives with residues His227, Arg276, Gln279, and Ser230. H-bond formation with the βI tubulin isotype shows differences at the residues level. Notably, the residues Lys19, Gln276, and Gln279 of the βI tubulin isotype play a predominant role in establishing hydrogen bonds. It is noticeable that the maximum hydrogen-bond distance is 3.5 Å, which corresponds to strong H bonds. Many more residues with significant interactions existed and were involved in hydrophobic interaction at the binding site of β-tubulin isotypes and ligands (Table 3). The results of interaction analysis showed the dominance of hydrophobic interactions to hydrogen bonding in this study.

**Figure 2:**
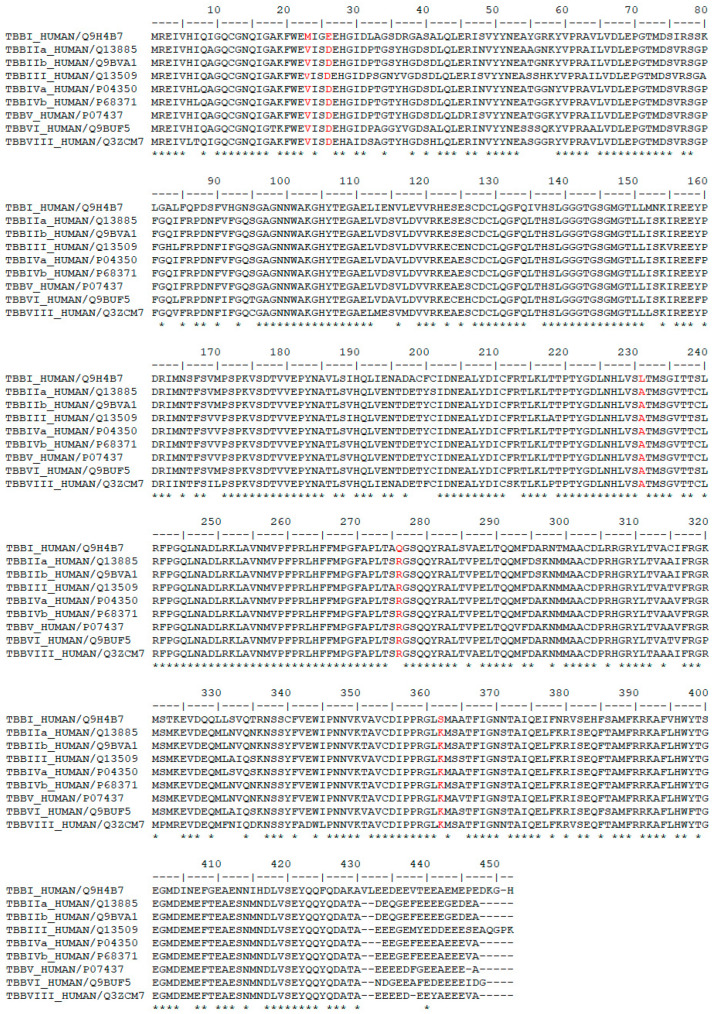
Multiple sequence alignment of human β-tubulin isotypes. The isotype βI shows variations of residues Arg276-Gln, Lys362-Ser, Val23-Met, Ala231-Leu, and Asp26-Glu at the griseofulvin binding pocket. Residue variations in the griseofulvin binding pocket are shown in red. Symbol ‘*’ demonstrates amino acid positions, which have a fully conserved amino acid residue.

We then investigated the residue composition changes in the griseofulvin binding pocket of different β-tubulin isotypes. The multiple sequence analysis study demonstrates residue variations in human β-tubulin isotypes at different locations (Figure 2). Griseofulvin shows differences in binding energy ranging from −6.8 to −7.38 kcal mol^−1^ with respect to the residue composition variations in and around the binding pocket of different β-tubulin isotypes. The griseofulvin binding pocket of βI has five residue changes, including Arg276-Gln, Lys362-Ser, Val23-Met, Ala231-Leu, and Asp26-Glu, compared to other β-tubulin isotypes. Here, Leu215, Lue217, and Cys211 interact hydrophobically with the methoxy group of griseofulvin. Afterwards, an analysis of the docking complex of βI-tubulin isotype–griseofulvin shows that methoxy and carbonyl groups of griseofulvin form hydrogen bonding interactions with residues Gln276 and Gln279, respectively. Thus, these results suggest that the residue composition variation in and around the griseofulvin binding pocket results in differences in binding energy, conformation, and hydrogen bonding interactions among the different β-tubulin isotype–griseofulvin complexes.

**Figure 3:**
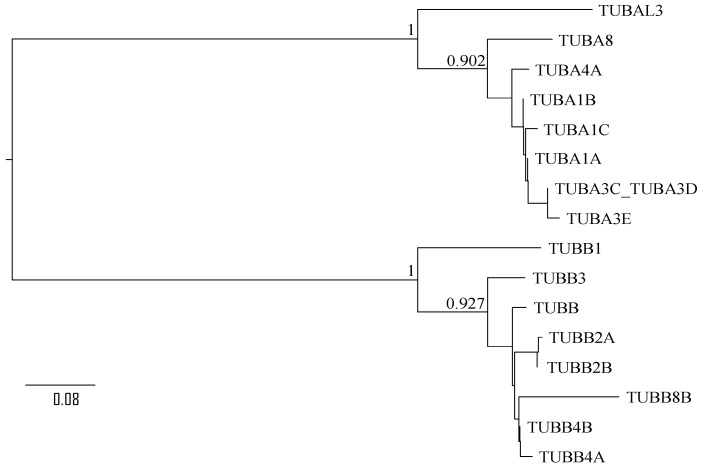
Phylogenetic tree from PhyML (LG substitution model and TLR option for simultaneous optimization of topology, branch length, and rates) based on aligned amino acid sequences of tubulin proteins. The tree was mid-point rooted.

Phylogenetic reconstruction with PhyML based on aligned amino acid sequences of α and β tubulin proteins (Figure 3) shows that βI (TUBB1) resulted from the earliest gene duplication within the β tubulin clade. This explains the sequence divergence of TUBB1 from the rest of the β tubulin proteins in Figure 2. The earliest lineage within the α tubulin clade is TUBAL3 (Figure 3). TUBB1 and TUBAL3 have the highest isoelectric point (*pI* = 4.93 and 5.98, respectively) among all the tubulin proteins in Figure 3, whereas the rapidly evolving TUBB8B has the smallest *pI* (=4.59) and features the smallest number of lysine residues. There is a tendency for more recently derived paralogues to have low *pI* values, consistent in both α and β clade (Figure 3), although the biological significance of this *pI* change is not obvious. All the tubulin proteins are expected to be negatively charged at normal cytoplasmic pH near 7 because their *pI* values are all smaller than 7.

The study was continued for compound G1 (CID 25171849) with the best binding energies and best interactions, in terms of hydrogenic and hydrophobic bonds towards all the β-tubulin isotypes for a better understanding of the effect of the β-tubulin isotypes interactions with a griseofulvin derivative. The structural model of the binding mode and conformation of compound G1 at the binding site of nine distinct β-tubulin isotypes are shown in Table 4.

### 2.2. Molecular Dynamic (MD) Simulations

Molecular docking results suggested that compound G1 (CID 25171849) serves as the best interacting compound with all the β-tubulin isotypes and, therefore, it was considered for the MD analysis. The cytotoxicity of this compound against the breast cancer cell line MDA-MB-231 has been shown by MTT colorimetric assay in a previous study [34]. The compound G1 showed the highest number of hydrogenic and hydrophobic interactions with the lowest free binding energies ranging from −8.65 to −9.32 kcal mol^−1^ (Table 5), corresponding to the highest affinity towards β-tubulin isotypes. To investigate the protein structure stability and conformational changes upon binding ligands, we performed molecular dynamic (MD) simulations over the β-tubulin-G1 docked complexes for 200 ns. The trajectory files from MD simulations were analyzed for root-mean-square deviation (RMSD), root-mean-square fluctuation (RMSF), radius of gyration (Rg), solvent-accessible surface area (SASA), and molecular mechanics Poisson-Boltzmann surface area (MM-PBSA).

**Figure 4:**
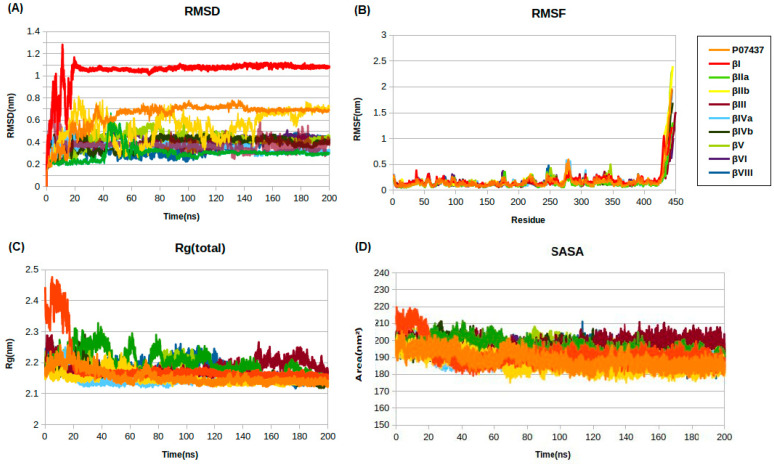
Structural dynamics of β-tubulin isotypes in complex with compound G1. (**A**) Root-mean-square deviation (RMSD), (**B**) root-mean-square fluctuations (RMSF), (**C**) radius of gyration (Rg), and (**D**), solvent-accessible surface area (SASA). P07437 corresponds to β-tubulin protein without ligand.

The root-mean-square deviations (RMSD) were calculated to examine the stability of the molecular dynamics simulation. The RMSD analysis complexes suggest that most β-tubulin–G1 complexes reached their equilibrium conformation after a time period of 100 ns and then, retained their stability during the simulation (Figure 4A). However, βIIb still seems to be fluctuating even after 180 ns from the RMSD graph in Figure 4A. It is notable that unfavorable interaction with His227 of G1 at tubulin βI resolved in the first few ns of the MD simulation; and then, a new conformation is stabilized, as also suggested by the high RMSD at this isotype. The average RMSD values were found to be the lowest for the G1 complexes with the βII (0.29 Å), followed by βIVa (0.32 Å) and βIII (0.37 Å) isotypes. No significant variations were observed in the average values of RMSFs, demonstrating the consistent complex exposure for a favorable conformational cavity of the β-tubulin proteins (Table 5). The average Rg values show that the packing of β-tubulin upon ligand binding is not considerably different from β-tubulin protein alone during the last 20 ns of MD simulations (Table 5). From the SASA plot in Figure 4D, we can see that the SASA values of almost all the β-tubulin–G1 complexes are greater overall than the β-tubulin without ligand, indicating the increases of solvent-accessible surface area and stability of the protein. The highest SASA value was observed in βIII (200.1 Å^2^), followed by βV (193.7Å^2^) and βIV (191.6 Å^2^), revealing the protein volume expansion and low variation throughout the simulation time (Table 5).

#### Molecular Mechanics Poisson-Boltzmann Surface Area (MM-PBSA)

MM/PBSA was originally defined by Kollman et al. [35], which can be used as a reliable and practical approach to predicting binding affinities. Using the last 30 ns steady RMSD trajectories, 1000 snapshots were taken at regular intervals to study the binding free energy upon binding, which is one of the most common techniques for calculating interaction energies of biomolecular complexes. Table 6 shows the calculated average values of the various binding energies, such as the van der Waals, electrostatic, polar solvation, and SASA energies. Our hit compound G1 showed the average free binding energy of −34.6 kcal mol^−1^, which is favorable for binding to β-tubulin isotypes. These findings confirm the docking analysis, indicating that compound G1 has a strong binding affinity towards β-tubulin isotypes, as seen in Table 6.

### 2.3. Tubulin Isotype Expression in Cancer

Clinical observations showed altered expression of tubulin isotypes in a range of cancers. These β-tubulins are encoded by multiple genes that are expressed tissue-specifically; e.g., βI is expressed in bone marrow and lymphoid tissue, βII in brain, βIII in neuronal and testicular cells, βIV in neuronal and glial cells, βVI in the erythroid cells and platelets, and βVIII is observed in testicular cells (Table 4). High expression of several β-tubulin isotypes, including I, II, III, IVa, and V tubulins, has been linked to aggressive clinical behavior, chemotherapy drug resistance, and poor patient outcome in many cancers, including lung, breast, ovarian, and gastric cancers. β-tubulin class III has been extensively investigated because of its role in resistance to antimitotic drugs. For example, it has been demonstrated that paclitaxel-resistant lung cancer cells and ovarian tumors have higher levels of β-tubulin classes III and IVa isotypes [36]. Tubulin isotypes βIVa and βIVb accounted for more than half of the β-tubulin in breast and colon cancer cells [37]. Detailed information on β-tubulin isotypes in cancer cells is provided in Table 7.

## 3. Computational Methodology

### 3.1. Ligand and Receptor Preparation

The 3D SDF file formats for griseofulvin (compound CID 441140), taxol (compound CID 36314), as well as 464 griseofulvin analogues, were retrieved from the PubChem database (https://pubchem.ncbi.nlm.nih.gov/). To have a comprehensive study, all the available data for griseofulvin derivatives from PubChem database were included in this study. The biological activity of some griseofulvin derivatives such as antifungal and anticancer activities was reported in previous studies; however, there is limited information on the biological activity of most derivatives recorded in PubChem. The CIDs and more details about 464 griseofulvin derivatives are listed in Supplementary File S1. The pdbqt ligand file was then preprocessed for docking by adding partial charges, atom types, and assigning the torsional flexibility of atoms to the ligand; in addition, their optimization was generated using Open Babel version 2.4.1.

Since there is no crystal structure available for some β-tubulin isotypes, homology modeling was performed for protein sequences of human Tubulin beta-5 chain (TUBB5) using MODELLERv18 [45]. This sequence was retrieved from UniProt (ID P07437) and then aligned against Protein Data Bank deposited sequences using BLAST. A protein of known tubulin structure (PDB ID: 6I2I) with the highest sequence identity to the target protein was chosen as a template for homology modeling. The template and the target file were used as input to MODELLER to align the sequence and to build the model structure. The best model was chosen based on the discrete optimized potential energy (DOPE) score. The energy minimization of the model structure was carried out using Chimera 1.15 software, and the quality of the protein structure was evaluated using PROCHECK by Ramachandran plot [46]. Evaluation of the model using Ramachandran plot showed that 87.1% of residues lie in favored regions, followed by 11.6% in allowed regions, indicating a good quality model (Supplementary File S2). In addition, the Ramachandran plot of the human Tubulin beta-5 chain (TUBB5) protein retrieved from AlphaFold was evaluated and the results showed that the number of residues present in the favoured region is 94.6%, and the allowed region is 4.9% (Supplementary File S3). This finding shows an agreement between the results of the structure generated with homology modeling and AlphaFold. Therefore, the nine different human β-tubulin isotypes proteins were retrieved from AlphaFold database for further analysis in this study [47]. The AlphaFold IDs of these β-tubulin sequences are as follows: βI (Q9H4B7), βIIa (Q13885), βIIb (Q9BVA1), βIII (Q13509), βIVa (P04350), βIVb (P68371), βV (P07437), βVI (Q9BUF5), and βVIII (Q3ZCM7). 

### 3.2. Sequence Analysis of β-Tubulin Isotypes

To identify the various residues at the griseofulvin binding pocket, the sequences of nine different human β-tubulin isotypes were aligned with MAFFT with the slow, but accurate, G-INS-i option [48].

### 3.3. Virtual Screening and In Silico Molecular Docking

PyRx 0.8 [49] from MGL Tools (https://ccsb.scripps.edu/mgltools/) was used for the virtual screening of griseofulvin analogues library molecules against β-tubulin, using default settings. In this study, TUBB5 (AlphaFold ID P07437) was used as a receptor for screening using AutoDock vina in PyRx. A grid box of size 23 Å × 25 Å × 26 Å with a spacing of 0.375 Å and co-ordinates x = 200.01, y = 268.74, and z = 334.49 was prepared at the β-tubulin. The five lowest-scoring analogues of griseofulvin, griseofulvin, and taxol were then docked into the β-tubulin isotypes binding sites using the Lamarckian genetic algorithm in AutoDock 4 software [50]. The geometric center of the grid box was placed at the macromolecule with coordinates x = −19.276, y = 9.865, and z = −1.569. A grid box of size 70 Å × 64 Å × 70 Å with a spacing of 0.375 Å was prepared at the β-tubulin to cover the putative griseofulvin binding site. The ligand with the lowest binding-free energies and the best modes of interactions with different β-tubulin binding sites was considered for further analysis. The interactions between the receptors and the ligands were visualized using Discovery Studio Visualizer v.20 (https://discover.3ds.com/discovery-studio-visualizer-download).

### 3.4. Molecular Dynamics Simulation

To validate the docking results, molecular dynamics (MDs) simulation was performed for the best derivative-docked complexes with nine different human β-tubulin isotypes; i.e., βI, βIIa, βIIb, βIII, βIVa, βIVb, αβV, βVI, and αβVIII, using the GROMACS-2022 software package [51] to carry out 200 ns simulations with an AMBER99SB force field. The Ante Chamber PYthon Parser interface (ACPYPE) was used to create the force field parameters for the ligand [52]. The TIP3P water model was selected for solvating complexes, and then sodium and chloride ions were added to neutralize the system. The conditions of periodic boundary (PBC) were used. Energy minimization was carried out at 1000 kJ/mol/nm. The system was equilibrated in the NVT and NPT ensembles for 1 ns. Sufficient amount of Na+ and Cl counter-ions were added to achieve the overall system charge neutrality and ionic strength of 0.15 M. To maintain the temperature and pressure at 310 K and 1.0 bar, the Nose-Hoover thermostat and the Berendsen barostat were used, respectively [53]. The trajectories were generated every 2 fs and saved every 2 ps. Post-MD analyses were performed; these included root-mean-square deviation (RMSD), root-mean-square fluctuations (RMSF), the radius of gyration (Rg), solvent-accessible surface area (SASA), and molecular mechanics Poisson-Boltzmann surface area (MM-PBSA).

## 4. Conclusions

The FDA-approved drug griseofulvin has shown the potential for suppressing cancer cell division and inducing cell death through interaction with the mitotic spindle microtubule [8], but its mechanism and interaction with tubulin molecules have remained poorly understood. In this study, sequence analysis, molecular docking, molecular dynamics simulations, and binding-free energy calculations were used to investigate the binding affinity of griseofulvin and its derivatives with nine human β-tubulin isotypes I, IIa, IIb, III, IVa, IVb, V, VI, and VIII. The human βI-tubulin isotype was found to have different residue compositions at the griseofulvin binding pocket, while no significant differences were found in other β-tubulin isotypes.

The results of molecular docking demonstrate that griseofulvin has a remarkable affinity toward β-tubulin isotypes, i.e., at the taxol binding site, as reported previously [27]. Griseofulvin exhibited different binding modes and binding energies for different β-tubulin isotypes; this could be due to changes in residue composition in and around the binding pocket of different β-tubulin isotypes. The residues Gln276 and Gln279 in βI-tubulin, and residue Thr274 in other β-tubulin isotypes are involved in the hydrogen bonding interactions with griseofulvin. Interestingly, our docking analysis showed that the binding energies of griseofulvin toward β-tubulin isotypes are close to the binding energies estimated for taxol. Further, the binding free-energy calculation revealed that derivatives G1-G5 have more negative binding energies ranging from −7.29 to −10.3 kcal/mol^−1^ and they could bind favorably to human β-tubulin isotypes even better than taxol with binding energies ranging from −6.29 to −8.11 kcal/mol^−1^.

The structure analysis of selected derivatives revealed functional groups such as methoxy, and aromatic ether groups in derivatives G1 and G2 provided better interactions and higher affinities toward receptors. Furthermore, structures containing benzene in derivatives G3 and G4, and the acetate ester group in derivative G5 enhanced the interactions between ligands and receptors.

Molecular dynamics simulations were performed on β-tubulin isotype–G1 docked complexes, to further investigate the structural stability and conformational changes of β-tubulin isotypes upon binding to ligands. Furthermore, β-tubulin isotypes IVb, followed by IIb and IIa, showed lower (more negative) binding energies with −30.8, −25.9, and −25.3 kcal mol^−1^ corresponding to more favorable interactions. From MM-PBSA, it is evident that compound G1 has the most favorable binding to the βI-tubulin isotype with a free-binding energy of −34.6 kcal mol^−1^. The residue changes at the binding site could be one of the reasons behind the more negative binding-free energy of the G1 derivative toward the βI-tubulin isotype. Similarly, a previous study investigated the differential binding affinity of an anti-mitotic agent colchicine analogue to different αβ-tubulin isotypes. They found the changed residues at the binding site of an αβIII isotype were responsible for affecting the binding affinity of a colchicine analogue [54]. Another interesting study explored the interaction of the microtubule-depolymerizing agent indanocine with different human αβ tubulin isotypes. Their findings suggested differences in amino acid sequences at the indanocine binding pockets of βI, βIIa, βIII, and βVI isotypes, which resulted in less binding-free energy toward indanocine [55].

One of the limitations of the present study is the using of AlphaFold β-tubulin isotypes protein structures to perform molecular docking analysis, as there were no available crystal structures for some β-tubulin isotypes. However, homology modeling was performed for one of these β-tubulin isotypes, and evaluation of the model using a Ramachandran plot showed a good agreement between the results of the structure generated with homology modeling and AlphaFold structure. The other limitation of the current study is that molecular dynamics simulations were performed only for the G1 derivative due to the time-consuming data analysis of molecular dynamics simulations and our limited computational resources, although other derivatives such as G2 showed good binding affinities and favorable interactions toward β-tubulin isotypes. Our current computational study provides a significant understanding of the involved molecular interactions of griseofulvin with human β-tubulin isotypes, which provides insight for designing potent griseofulvin analogues for specific tubulin isotypes. These superior analogues may improve the treatment of patients with advanced carcinoma that demonstrate tubulin isotype specificity, as well as in the development of innovative new cancer medicines in the future.

## Figures and Tables

**Table 1:** Chemical two-dimensional (2D) structures of selected derivatives as well as computed binding energies towards TUBB5, and also estimated Moriguchi octanol-water partition coefficient (MlogP).

PubChem IDs	Structures	Binding Energies (kcal/mol)	MlogP
441140 (griseofulvin)	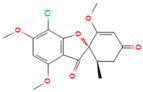	−6.6	0.71
CID 25171849 (G1)	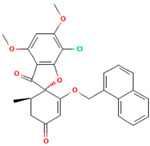	−9.1	2.43
CID 46844082 (G2)	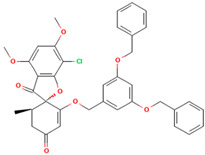	−9.0	2.98
CID 73331205 (G3)	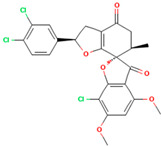	−9.1	2.95
CID 73331488 (G4)	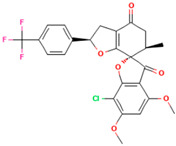	−9.3	2.51
CID 118263246 (G5)	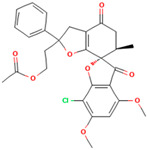	−8.8	1.96

**Table 2:** Heat map of binding energies interactions between taxol, griseofulvin, and the five griseofulvin derivatives and nine distinct β-tubulin isotypes. Red colour indicates areas with the lowest binding energies and green indicates the highest binding energies.

	AlphaFold ID	Taxol	Griseofulvin	25171849 (G1)	46844082 (G2)	73331205 (G3)	73331488 (G4)	118263246 (G5)
BI	Q9H4B7	−6.29	−6.8	−8.7	−7.29	−8.09	−7.36	−7.95
BIIa	Q13885	−6.95	−7.38	−8.89	−9.07	−8.91	−8.21	−8.31
BIIb	Q9BVA1	−7.17	−7.1	−8.8	−10.3	−8.7	−8.1	−8.3
BIII	Q13509	−6.43	−7.38	−8.72	−8.75	−8.96	−8.31	−8.41
BIVa	P04350	−8.11	−7.24	−9.04	−9.88	−8.75	−8.03	−8.37
BIVb	P68371	−7.71	−7.24	−8.65	−9.68	−8.84	−7.95	−8.23
BV	P07437	−7.6	−6.9	−9.32	−9.16	−8.67	−7.6	−8.72
BVI	Q9BUF5	−7.66	−7.24	−8.65	−9.63	−9.11	−8.1	−8.41
BVIII	Q3ZCM7	−7.25	−7.29	−8.8	−9.12	−9.04	−8.17	−8.31
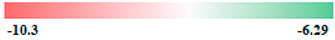								

**Table 3:** Binding energy as well as interactions detail of ligands with different human β-tubulin isotypes after molecular docking.

β-Tubulin Isotype	Ligands	Binding Energy (kcal/mol)	H bonds	Hydrophobic Interaction
βI	Taxol	−6.29	Lys19, Gln276	Leu215, His227
	Griseofulvin	−6.8	Gln276, Gln279	Cys211, Leu215, Leu217, His227
	G1	−8.7	Gln279	Met23, Leu215, Leu217, His227, Leu231, Pro358, Leu361
	G2	−7.29	Gln279	Met23, Leu215, Leu217, Pro272, Thr274, Gln276, Gln279, Gly360, Leu261
	G3	−8.09	Lys19	Lys19, Met23, Leu215, Gly223, His227, Leu228, Leu231, Phe270, Leu273,
	G4	−7.36	Lys19	Met23, Lys19, Leu215, Gly223, His227, Leu228, Leu231, Phe270, Pro272, Leu273
	G5	−7.95	Gln276, Gln279	Leu215, Leu217, His227, Thr274
βIIa	Taxol	−6.95	Gln279, Arg359, Lys362	Pro272, Gln280, Leu284, Leu361
	Griseofulvin	−7.38	Thr274	Leu215, His227, Arg359, Arg276, Gln279
	G1	−8.89	Thr274	Leu215, His227, Thr274, Arg276, Leu361
	G2	−9.07	-	Val23, Leu215, Leu361, Lys362, Arg359
	G3	−8.91	Thr274	Leu215, His227, Arg276, Gly360
	G4	−8.21	Thr274	Leu215, His227, Arg276, Gly360
	G5	−8.31	Thr274, Gln279	Leu215, Arg276, Thr274, Gln279
βIIb	Taxol	−7.17	His227, Gln279	Val23, Leu215, Ala231, Pro272, Arg276, Gln279, Arg359, Leu361
	Griseofulvin	−7.1	Thr274	Leu215, His227, Gln279, Arg276
	G1	−8.8	Thr274	Leu215, His227, Arg276, Leu361
	G2	−10.3	Thr274	Leu215, His227, Thr274, Arg276, Gln279, Arg282, Leu284, Arg359, LeU361
	G3	−8.7	Thr274	Leu215, His227, Thr274, Arg276, Gly360
	G4	−8.1	Thr274	Leu215, His227, Thr274, Arg276, Gly360
	G5	−8.3	Thr274, Gln279	Leu215, His227, Thr274, Arg276
βIII	Taxol	−6.43	His227, Arg276, Gly360	Leu215, His227, Pro272, Arg276, Gln279, Gly360, Leu361
	Griseofulvin	−7.38	Thr274	Leu215, His227, Arg276, Gln279
	G1	−8.72	-	Leu215, Thr274, His227, Leu273, Arg276, Gly360, Leu361
	G2	−8.75	Thr274	Asp26, Val23, Leu215, His227, Leu361, Leu273, Arg276, Arg359
	G3	−8.96	Thr274	Leu215, His227, Thr274, Arg276, Gly360, Leu361
	G4	−8.31	Thr274	Leu215, His227, Arg276, Gln279, Gly360
	G5	−8.41	Thr274, Gln279	Leu215, Leu217, Arg276, His227
βIVa	Taxol	−8.11	Lys19, Thr274, Arg359	Leu215, Leu217, His227, Leu228, Ala231, Phe270, Leu273, Pro358, Gly360,
	Griseofulvin	−7.24	Thr274	Leu215, His227, Pro272, Arg276, Gln279, Leu361
	G1	−9.04	-	Leu215, His227, Thr274, Arg276, Leu361
	G2	−9.88	Thr274	Leu15, Arg276, Leu284
	G3	−8.75	Gln279	Val23, Leu215, Gly223, Asp224, His227, Leu228, Ala231, Pro358, Arg359, Leu361
	G4	−8.03	-	Leu215, His227, Arg276, Gly360
	G5	−8.37	Thr274	Leu215, Leu217, His227, Arg276, Thr274
βIVb	Taxol	−7.71	Lys19, Thr274, Arg359	Leu215, His227, Leu228, Ala231, Leu273, Leu300, Pro358
	Griseofulvin	−7.24	Thr274	Leu215, His227, Arg276, Gln279
	G1	−8.65	Thr274	Leu215, His227, Arg276, Leu361
	G2	−9.68	Thr274	Val23, Leu215, His227, Pro272, Arg276, Pro358, Arg359, Gly360, Leu361
	G3	−8.84	Gln279	Val23, Leu215, Gly223, Asp224, His227, Ala231, Pro358, Arg359, Leu361
	G4	−7.95	Thr274	Leu215, Leu217, His227, Thr274, Arg276, Gly360
	G5	−8.23	Thr274	Leu215, Leu217, His227, Thr274, Arg276
βV	Taxol	−7.6	His227, Arg276, Gln279, Arg359	Val23, Leu215, His227, Ala231, Arg359
	Griseofulvin	−6.9	Thr274	Leu215, His227, Thr274, Arg276, Gln279, Arg359
	G1	−9.32	Ser230	Lys19, Val23, His227, Ala231, Pro272, Arg359, Leu361
	G2	−9.16	Gln279	Val23, Leu215, Ala231, Phe270, Leu273, Thr274, Arg276, Gln279, Arg359, Leu361,
	G3	−8.67	His227	Lys19, Val23, Leu215, His227, Leu228, Ala231, Arg359, Leu273
	G4	−7.6	His227, Ser230	LyS19, Val23, Leu215, His227, Leu228, Ala231, Leu273, Arg359
	G5	−8.72	Ser230	Lys19, Val23, His227. Pro272, Arg259, Arg359, Leu361,
βVI	Taxol	−7.66	Lys19, Arg276, Arg359	Val23, Asp224, His227, Ala231, Phe270, Arg359
	Griseofulvin	−7.24	Thr274	Leu215, His227, Arg276, Gln279
	G1	−8.65	Arg276, Gln279	Val23, Asp224, His227, Ala231, Pro358, Arg359, Leu361
	G2	−9.63	-	Lys19, Glu22, Val23, Asp26, Leu215, Leu217, His227, Arg359, Leu273, Arg276, Gln279, Leu361
	G3	−9.11	Gln279	Val23, Cys211, Leu215, Gly223, Asp224, His227, Ala231, Pro358, Arg359, Leu361
	G4	−8.1	Gln279, Arg359	Val23, Glu27, Leu215, Asp224, His227, Leu228, Ala231, Pro358, Arg359, Leu361
	G5	−8.41	Thr274	Leu215, His227, Thr274, Arg276
βVIII	Taxol	−7.25	Arg276, Arg359	Lys19, Glu22, Val23, Asp26, His227, Phe270, Arg359
	Griseofulvin	−7.29	Thr274	Leu215, Arg276, His227, Thr274, Arg276, Arg359,
	G1	−8.8	Thr274	Leu215, His227, Thr274, Arg276, Leu361
	G2	−9.12	Thr274	Leu215, His227, Arg276, Gln279, Leu284,
	G3	−9.04	Gln279	Val23, LeU215, His227, Leu228, Ala231, Pro358, Arg359, Leu361
	G4	−8.17	Thr274	Leu215, His227, Thr274, Arg276, Gly360, Leu361
	G5	−8.31	Thr274	Leu215, His227, Thr274, Arg276

**Table 4:** The 3D- and 2D-binding conformations and active residues involved in interactions between compound G1 and nine distinct β-tubulin isotypes.

Β-Tubulin Isotype	Binding Energy (kcal/mol)	3D Interactions	2D Interactions
βI	−8.7	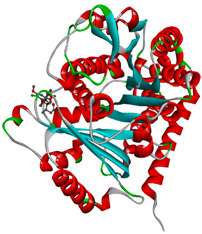	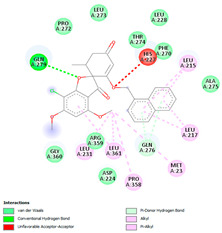
βIIa	−8.89	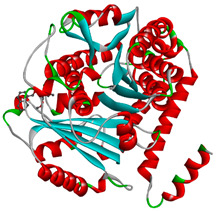	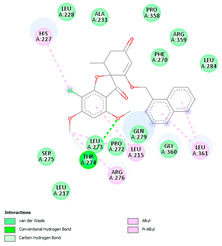
βIIb	−8.8	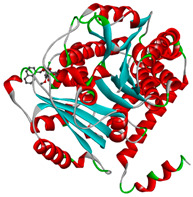	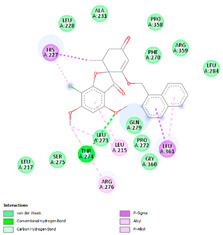
βIII	−8.72	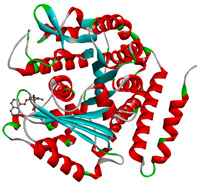	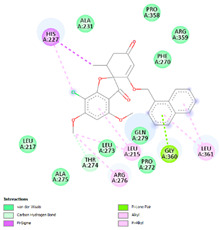
βIVa	−9.04	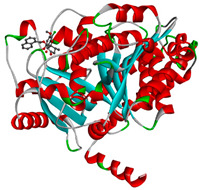	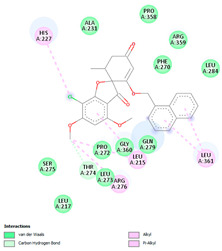
βIVb	−8.65	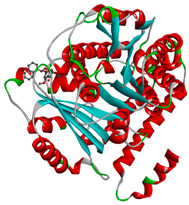	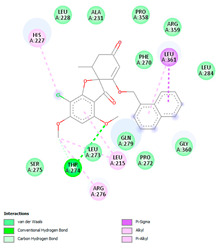
βV	−9.32	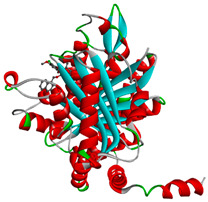	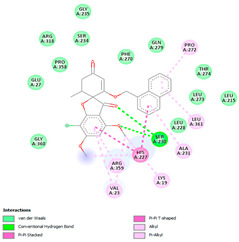
βVI	−8.65	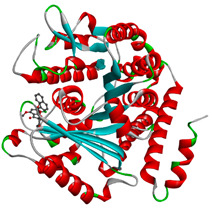	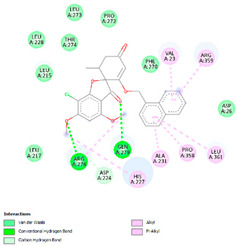
βVIII	−8.8	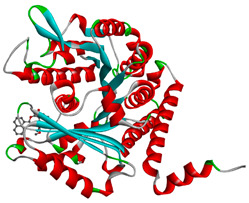	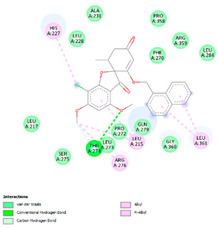

**Table 5:** The average of RMSD, RMSF, Rg, and SASA for nine distinct β-tubulin isotypes during the last 20 ns of MD simulations.

Complex	RMSD ± SD (Å)	Rg ± SD (Å)	RMSF ± SD (Å)	SASA ± SD (Å^2^)
P07437	0.68 ± 0.008	2.13 ± 0.04	0.096 ± 0.05	185.5 ± 2.26
βI-G1	1.08 ± 0.08	2.15 ± 0.05	0.09 ± 0.05	188.1 ± 2.7
βIIa-G1	0.29 ± 0.008	2.14 ± 0.05	0.096 ± 0.04	183.4 ± 2.2
βIIb-G1	0.70 ± 0.02	2.14 ± 0.08	0.108 ± 0.101	190.3 ± 2.8
βIII-G1	0.37 ± 0.04	2.15 ± 0.18	0.155 ± 0.201	200.1 ± 3.1
βIVa-G1	0.32 ± 0.006	2.19 ± 0.05	0.093 ± 0.047	188.1 ± 2.5
βIVb-G1	0.38 ± 0.022	2.15 ± 0.06	0.101 ± 0.074	188.7 ± 2.6
βV-G1	0.41 ± 0.02	2.13 ± 0.09	0.11 ± 0.07	193.7 ± 2.7
βVI-G1	0.43 ± 0.01	2.14 ± 0.04	0.097 ± 0.047	191.6 ± 2.5
βVIII-G1	0.44 ± 0.01	2.13 ± 0. 04	0.094 ± 0.063	185.8 ± 2.3

**Table 6:** Averaged binding free energies of the simulated β-tubulin–G1 complexes from MM-PBSA estimation during the last 30 ns.

Complex	van der Waal Energy (kcal/mol)	Electrostatic Energy (kcal/mol)	Polar Solvation Energy (kcal/mol)	SASA Energy (kcal/mol)	Binding Energy (kcal/mol)
βI-G1	−40.0 ± 2.7	−4.5 ± 1.4	21.1 ± 2.5	−4.0 ± 0.3	−34.6 ± 3.0
βIIa-G1	−41.0 ± 3.4	−0.43 ± 0.1	19.9 ± 3.6	−4.6 ± 0.3	−25.3 ± 13.2
βIIb-G1	−40.1± 3.7	−0.3 ± 0.1	18.6 ± 3.0	−4.1 ± 0.3	−25.9 ± 3.1
βIII-G1	−35.2 ± 7.6	−0.7 ± 0.3	19.2 ± 3.7	−3.8 ± 0.6	−20.3 ± 6.5
βIVa-G1	−43.2± 3.1	−4.3 ± 2.1	27.6 ± 4.4	−4.4 ± 0.3	−24.3 ± 3.0
βIVb-G1	−51.3 ± 3.0	−3.4 ± 1.5	30.0 ± 3.6	−5.1± 0.3	−30.8 ± 3.6
βV-G1	−40.7 ± 6.1	−0.9 ± 0.3	20.4 ± 4.1	−4.2 ± 0.4	−25.2 ± 4.4
βVI-G1	−40.5 ± 3.1	−3.6 ± 1.4	23.3 ± 2.7	−4.2 ± 0.3	−25.0 ± 3.1
βVIII-G1	−35.9± 3.5	−1.2 ± 0.5	18.3 ± 2.0	−3.8 ± 0.3	−22.5 ± 2.9

**Table 7:** Clinical studies of β-tubulin isotypes in cancer cells.

β-Tubulin Isotype	Tissue Specificity	Single-Cell Type Expression Cluster	Cell-Line Enriched	Tumor Type	Cancer Prognostic and Effect	Reference
βI	Bone marrow, lymphoid tissue	Myeloid cells	HEL	Breast cancer	Poor response to docetaxel treatment	[22,38]
βIIa	Brain	Squamous epithelial cells, keratinization	SuSa	Breast and ovarian cancer	Prognostic marker in urothelial cancer (unfavorable) and renal cancer (favorable); poor response to taxane treatment or advanced stage disease	[38,39,40]
βIIb	Brain, choroid plexus	Muller glia cells, visual perception	SCLC-21H	Breast and ovarian cancer	Prognostic marker in endometrial cancer (unfavorable); poor response to taxane treatment	[38,39,40]
βIII	Brain, neuropeptide signaling	Pancreatic cells, digestion	SH-SY5Y	Breast, ovarian, prostate, uterine serous carcinoma cancer	Gene product is not prognostic; poor response to taxane treatment	[22,23,38,39,41,42,43]
βIVa	Brain, nervous system	Oligodendrocytes, myelin sheath organization	AN3-CA	Ovarian cancer	Prognostic marker in endometrial cancer (unfavorable); poor response to taxol treatment	[36,38]
βIVb	Ciliated cells, cilium, and cell projection	Mitochondria	MCF-7	Ovarian cancer	Prognostic marker in thyroid cancer and endometrial cancer (favorable), and liver cancer (unfavorable)	[36,38]
βV	Lymphoid tissue, immune response regulation	Non-specific	WM-115	Non-small cell lung cancer (NSCLC)	Prognostic marker in renal cancer and liver cancer (unfavorable); favorable response to taxane treatment	[38,44]
βVI	Fibroblasts, ECM organization	Extravillous trophoblasts	TIME	Not specified	Prognostic marker in renal cancer (unfavorable) and urothelial cancer (unfavorable)	[38]
βVIII	Testis	Spermatogonia and spermatocytes	MCF-7	Not specified	Gene product is not prognostic	[38]

## Data Availability

Not applicable.

## Supplementary Materials

The following supporting information can be downloaded at: https://www.mdpi.com/article/10.3390/molecules28052384/s1, Table S1: Griseofulvin derivatives, Figure S1: Ramachandran plot analysis of modeled Human β-tubulin (TBB5_HUMAN) using MODELLER, Table S2: Ramachandran plot statistics of modeled Human β-tubulin using MODELLER, Figure S2: Ramachandran plot analysis of Human β-tubulin (TBB5_HUMAN) obtained from AlphaFold, Table S3: Ramachandran plot statistics of Human β-tubulin obtained from AlphaFold, Table S4: Virtual screening by PyRx.

## References

[1] Oxford A.E., Raistrick H., Simonart P. (1939). Studies in the Biochemistry of Micro-Organisms: Griseofulvin, C17h17o6cl, a Metabolic Product of *Penicillium griseo-fulvum* Dierckx. Biochem. J..

[2] Lambert D.R., Siegle R.J., Camisa C. (1989). Griseofulvin and Ketoconazole in the Treatment of Dermatophyte Infections. Int. J. Dermatol..

[3] Chooi Y.H., Cacho R., Tang Y. (2010). Identification of the Viridicatumtoxin and Griseofulvin Gene Clusters from *Penicillium aethiopicum*. Chem. Biol..

[4] Lee H.B., Mun H.Y., Nguyen T.T.T., Kim J.-C., Stone J.K. (2016). *Abieticola koreana* Gen. Et Sp. Nov., a Griseofulvin-Producing Endophytic *Xylariaceous ascomycete* from Korea. Mycotaxon.

[5] Ribeiro A.I., Costa E.S., Thomasi S.S., Brandão D.F.R., Vieira P.C., Fernandes J.B., Rossi Forim M., Ferreira A.G., Florentino Pascholati S., Pascholati Gusmão L.F. (2018). Biological and Chemical Control of Sclerotinia Sclerotiorum Using Stachybotrys Levispora and Its Secondary Metabolite Griseofulvin. J. Agric. Food Chem..

[6] Mead M.E., Raja H.A., Steenwyk J.L., Knowles S.L., Oberlies N.H., Rokas A. (2019). Draft Genome Sequence of the Griseofulvin-Producing Fungus *Xylaria flabelliformis* Strain G536. Microbiol. Resour. Announc..

[7] Aris P., Yan L., Wei Y., Chang Y., Shi B., Xia X. (2021). Conservation of Griseofulvin Genes in the Gsf Gene Cluster among Fungal Genomes. G3.

[8] Rebacz B., Larsen T.O., Clausen M.H., Rønnest M.H., Löffler H., Ho A.D., Krämer A. (2007). Identification of Griseofulvin as an Inhibitor of Centrosomal Clustering in a Phenotype-Based Screen. Cancer Res..

[9] Rathinasamy K., Jindal B., Asthana J., Singh P., Balaji P.V., Panda D. (2010). Griseofulvin Stabilizes Microtubule Dynamics, Activates P53 and Inhibits the Proliferation of Mcf-7 Cells Synergistically with Vinblastine. BMC Cancer.

[10] Aris P., Wei Y., Mohamadzadeh M., Xia X. (2022). Griseofulvin: An Updated Overview of Old and Current Knowledge. Molecules.

[11] Knasmüller S., Parzefall W., Helma C., Kassie F., Ecker S., Schulte-Hermann R. (1997). Toxic Effects of Griseofulvin: Disease Models, Mechanisms, and Risk Assessment. Crit. Rev. Toxicol..

[12] Zimmerman H.J. (1999). Hepatotoxicity: The Adverse Effects of Drugs and Other Chemicals on the Liver.

[13] Moseley R.H. (2013). Hepatotoxicity of Antimicrobials and Antifungal Agents. Drug-Induced Liver Disease.

[14] Jin H., Yamashita A., Maekawa S., Yang P., He L., Takayanagi S., Wakita T., Sakamoto N., Enomoto N., Ito M. (2008). Griseofulvin, an Oral Antifungal Agent, Suppresses Hepatitis C Virus Replication in Vitro. Hepatol. Res..

[15] Aris P., Mohamadzadeh M., Wei Y., Xia X. (2022). In Silico Molecular Dynamics of Griseofulvin and Its Derivatives Revealed Potential Therapeutic Applications for COVID-19. Int. J. Mol. Sci..

[16] Perez E.A. (2009). Microtubule Inhibitors: Differentiating Tubulin-Inhibiting Agents Based on Mechanisms of Action, Clinical Activity, and Resistance. Mol. Cancer Ther..

[17] Morris P.G., Fornier M.N. (2008). Microtubule Active Agents: Beyond the Taxane Frontier. Clin. Cancer Res..

[18] Verdier-Pinard P., Pasquier E., Xiao H., Burd B., Villard C., Lafitte D., Miller L.M., Angeletti R.H., Horwitz S.B., Braguer D. (2009). Tubulin Proteomics: Towards Breaking the Code. Anal. Biochem..

[19] Janke C. (2014). The Tubulin Code: Molecular Components, Readout Mechanisms, and Functions. J. Cell Biol..

[20] Nogales E., Wolf S.G., Downing K.H. (1998). Erratum: Structure of the Aβ Tubulin Dimer by Electron Crystallography. Nature.

[21] Vilmar A., Garcia-Foncillas J., Huarriz M., Santoni-Rugiu E., Sorensen J.B. (2012). Rt-Pcr Versus Immunohistochemistry for Correlation and Quantification of Ercc1, Brca1, Tubb3 and Rrm1 in Nsclc. Lung Cancer.

[22] Hasegawa S., Miyoshi Y., Egawa C., Ishitobi M., Taguchi T., Tamaki Y., Monden M., Noguchi S. (2003). Prediction of Response to Docetaxel by Quantitative Analysis of Class I and Iii Β-Tubulin Isotype Mrna Expression in Human Breast Cancers. Clin. Cancer Res..

[23] Roque D.M., Bellone S., English D.P., Buza N., Cocco E., Gasparrini S., Bortolomai I., Ratner E., Silasi D.-A., Azodi M. (2013). Tubulin-Β-Iii Overexpression by Uterine Serous Carcinomas Is a Marker for Poor Overall Survival after Platinum/Taxane Chemotherapy and Sensitivity to Epothilones. Cancer.

[24] Derry W.B., Wilson L., Khan I.A., Ludueña R.F., Jordan M.A. (1997). Taxol Differentially Modulates the Dynamics of Microtubules Assembled from Unfractionated and Purified Β-Tubulin Isotypes. Biochemistry.

[25] Banerjee A., Luduena R.F. (1992). Kinetics of Colchicine Binding to Purified Beta-Tubulin Isotypes from Bovine Brain. J. Biol. Chem..

[26] Xu K., Schwarz P.M., Ludueña R.F. (2002). Interaction of Nocodazole with Tubulin Isotypes. Drug Dev. Res..

[27] Crounse R.G. (1963). Effective Use of Griseofulvin. Arch. Dermatol..

[28] Liéby-Muller F., Le Baliner Q.H., Grisoni S., Fournier E., Guilbaud N., Marion F. (2015). Synthesis and Activities towards Resistant Cancer Cells of Sulfone and Sulfoxide Griseofulvin Derivatives. Bioorganic Med. Chem. Lett..

[29] Rønnest M.H., Rebacz B., Markworth L., Terp A.H., Larsen T.O., Krämer A., Clausen M.H. (2009). Synthesis and Structure−Activity Relationship of Griseofulvin Analogues as Inhibitors of Centrosomal Clustering in Cancer Cells. J. Med. Chem..

[30] Kellenberger E., Rodrigo J., Muller P., Rognan D. (2004). Comparative Evaluation of Eight Docking Tools for Docking and Virtual Screening Accuracy. Proteins: Struct. Funct. Bioinform..

[31] Trott O., Olson A.J. (2010). Autodock Vina: Improving the Speed and Accuracy of Docking with a New Scoring Function, Efficient Optimization, and Multithreading. J. Comput. Chem..

[32] Moriguchi I., Hirono S., Liu Q., Nakagome I., Matsushita Y. (1992). Simple Method of Calculating Octanol/Water Partition Coefficient. Chem. Pharm. Bull..

[33] Devalapally H., Chakilam A., Amiji M.M. (2007). Role of Nanotechnology in Pharmaceutical Product Development. J. Pharm. Sci..

[34] Rønnest M.H., Raab M.S., Anderhub S., Boesen S., Krämer A., Larsen T.O., Clausen M.H. (2012). Disparate Sar Data of Griseofulvin Analogues for the Dermatophytes Trichophyton Mentagrophytes, T. Rubrum, and Mda-Mb-231 Cancer Cells. J. Med. Chem..

[35] Kollman P.A., Massova I., Reyes C., Kuhn B., Huo S., Chong L., Lee M., Lee T., Duan Y., Wang W. (2000). Calculating Structures and Free Energies of Complex Molecules: Combining Molecular Mechanics and Continuum Models. Acc. Chem. Res..

[36] Kavallaris M., Kuo D.Y., Burkhart C.A., Regl D.L., Norris M., Haber M., Horwitz S.B. (1997). Taxol-Resistant Epithelial Ovarian Tumors Are Associated with Altered Expression of Specific Beta-Tubulin Isotypes. J. Clin. Investig..

[37] Hiser L., Aggarwal A., Young R., Frankfurter A., Spano A., Correia J.J., Lobert S. (2006). Comparison of Β-Tubulin Mrna and Protein Levels in 12 Human Cancer Cell Lines. Cell Motil. Cytoskelet..

[38] Uhlen M., Oksvold P., Fagerberg L., Lundberg E., Jonasson K., Forsberg M., Zwahlen M., Kampf C., Wester K., Hober S. (2010). Towards a Knowledge-Based Human Protein Atlas. Nat. Biotechnol..

[39] Ohishi Y., Oda Y., Basaki Y., Kobayashi H., Wake N., Kuwano M., Tsuneyoshi M. (2007). Expression of Beta-Tubulin Isotypes in Human Primary Ovarian Carcinoma. Gynecol. Oncol..

[40] Bernard-Marty C., Treilleux I., Dumontet C., Cardoso F., Fellous A., Gancberg D., Bissery M.-C., Paesmans M., Larsimont D., Piccart M.J. (2002). Microtubule-Associated Parameters as Predictive Markers of Docetaxel Activity in Advanced Breast Cancer Patients: Results of a Pilot Study. Clin. Breast Cancer.

[41] Levallet G., Bergot E., Antoine M., Creveuil C., Santos A.O., Beau-Faller M., De Fraipont F., Brambilla E., Levallet J., Morin F. (2012). High Tubb3 Expression, an Independent Prognostic Marker in Patients with Early Non–Small Cell Lung Cancer Treated by Preoperative Chemotherapy, Is Regulated by K-Ras Signaling Pathwayk-Ras and Tubb3 in Early Nsclc. Mol. Cancer Ther..

[42] Aoki D., Oda Y., Hattori S., Taguchi K.-I., Ohishi Y., Basaki Y., Oie S., Suzuki N., Kono S., Tsuneyoshi M. (2009). Overexpression of Class Iii Β-Tubulin Predicts Good Response to Taxane-Based Chemotherapy in Ovarian Clear Cell Adenocarcinoma. Clin. Cancer Res..

[43] Tsourlakis M.C., Weigand P., Grupp K., Kluth M., Steurer S., Schlomm T., Graefen M., Huland H., Salomon G., Steuber T. (2014). Βiii-Tubulin Overexpression Is an Independent Predictor of Prostate Cancer Progression Tightly Linked to Erg Fusion Status and Pten Deletion. Am. J. Pathol..

[44] Christoph D.C., Kasper S., Gauler T.C., Loesch C., Engelhard M., Theegarten D., Poettgen C., Hepp R., Peglow A., Loewendick H. (2012). Βv-Tubulin Expression Is Associated with Outcome Following Taxane-Based Chemotherapy in Non-Small Cell Lung Cancer. Br. J. Cancer.

[45] Eswar N., Eramian D., Webb B., Shen M.Y., Sali A. (2008). Protein Structure Modeling with Modeller. Structural Proteomics: High-throughput Methods.

[46] Laskowski R.A., MacArthur M.W., Moss D.S., Thornton J.M. (1993). Procheck: A Program to Check the Stereochemical Quality of Protein Structures. J. Appl. Crystallogr..

[47] Jumper J., Evans R., Pritzel A., Green T., Figurnov M., Ronneberger O., Tunyasuvunakool K., Bates R., Žídek A., Potapenko A. (2021). Highly Accurate Protein Structure Prediction with Alphafold. Nature.

[48] Katoh K., Asimenos G., Toh H., Posada D. (2009). Bioinformatics for DNA Sequence Analysis. Methods Mol. Biol..

[49] Dallakyan S., Olson A.J. (2015). Small-Molecule Library Screening by Docking with Pyrx. Chemical Biology.

[50] Morris G.M., Huey R., Lindstrom W., Sanner M.F., Belew R.K., Goodsell D.S., Olson A.J. (2009). Autodock4 and Autodocktools4: Automated Docking with Selective Receptor Flexibility. J. Comput. Chem..

[51] Bauer P., Hess B., Lindahl E. (2022). Gromacs 2022 Source Code. https://zenodo.org/record/6103835#.ZAHm33bMJPY.

[52] Sousa da Silva A.W., Vranken W.F. (2012). Acpype-Antechamber Python Parser Interface. BMC Res. Notes.

[53] Berendsen H.J.C., Postma J.P.M., Van Gunsteren W.F., DiNola A., Haak J.R. (1984). Molecular Dynamics with Coupling to an External Bath. J. Chem. Phys..

[54] Kumbhar B.V., Borogaon A., Panda D., Kunwar A. (2016). Exploring the Origin of Differential Binding Affinities of Human Tubulin Isotypes Aβii, Aβiii and Aβiv for Dama-Colchicine Using Homology Modelling, Molecular Docking and Molecular Dynamics Simulations. PLoS ONE.

[55] Kumbhar B.V., Panda D., Kunwar A. (2018). Interaction of Microtubule Depolymerizing Agent Indanocine with Different Human Aβ Tubulin Isotypes. PLoS ONE.

