# Supplementary material for: In Silico Exploration of Microtubule Agent Griseofulvin and Its Derivatives Interactions with Different Human β-Tubulin Isotypes
Source: Molecules. 2023 Mar 5;28(5):2384. doi: 10.3390/molecules28052384 (PMC10005519; doi:10.3390/molecules28052384)
Supplement: Supplementary file 1 [file molecules-28-02384-s001.zip › Figure S1, Table S2.pdf]

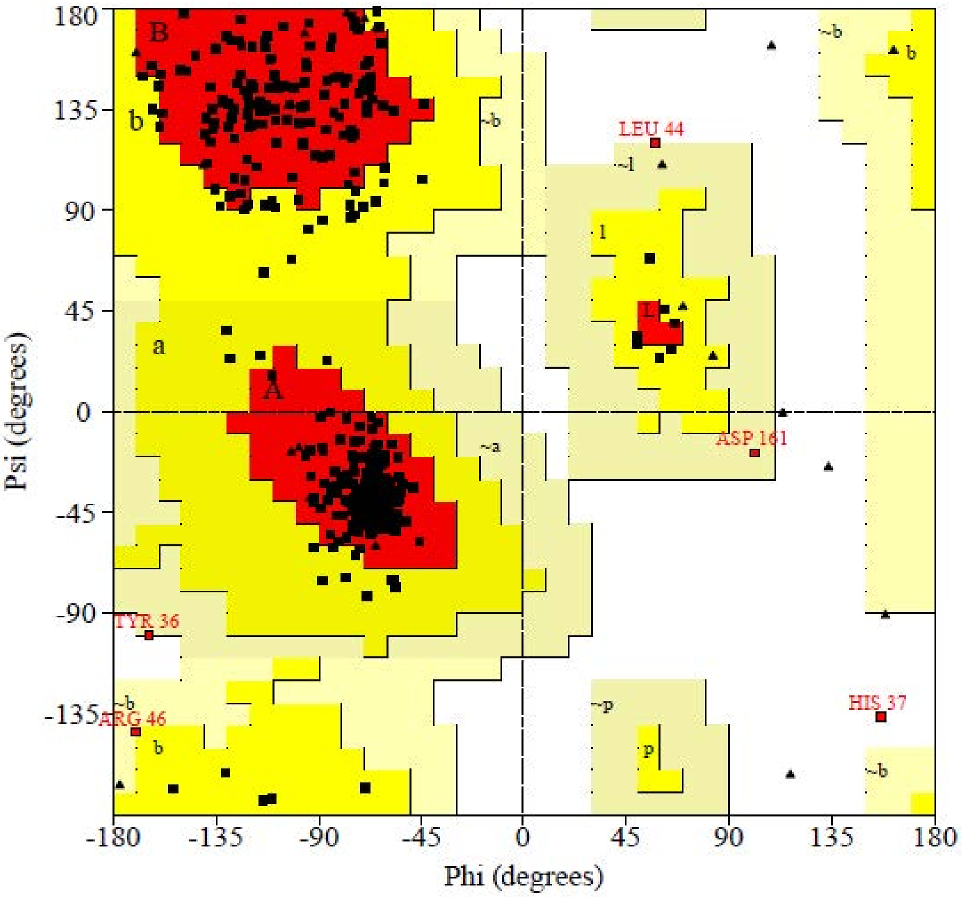

Figure S1. Ramachandran plot analysis of modeled Human β-tubulin (TBB5_HUMAN) using MODELLER. Here, red region indicates favored region, yellow region for allowed and light yellow shows generously allowed region and white for disallowed region. Phi and Psi angels determine torsion angels.

Table S2. Ramachandran plot statistics of modeled Human β-tubulin using MODELLER.

Plot statistics		
Residues in most favoured regions [A,B,L]	337	87.1%
Residues in additional allowed regions [a,b,l,p]	45	11.6%
Residues in generously allowed regions [~a,~b,~l,~p]	4	1.0%
Residues in disallowed regions	1	0.3%
	----	------
Number of non-glycine and non-proline residues	387	100.0%
Number of end-residues (excl. Gly and Pro)	2	
Number of glycine residues (shown as triangles)	35	
Number of proline residues	20	

Total number of residues	444	
